# Recyclable Magnesium-Modified Biochar Beads for Efficient Removal of Phosphate from Wastewater

**DOI:** 10.3390/nano13060966

**Published:** 2023-03-07

**Authors:** Biao Hu, Nina Yan, Zhiyu Zheng, Lei Xu, Hongde Xie, Jingwen Chen

**Affiliations:** 1 College of Chemistry, Chemical Engineering and Materials Science, Soochow University, Suzhou 215123, China; 2 Key Laboratory for Protected Agricultural Engineering in the Middle and Lower Reaches of Yangtze River, Institute of Agricultural Facilities and Equipment, Jiangsu Academy of Agricultural Sciences, Ministry of Agriculture and Rural Affairs, Nanjing 210014, China

**Keywords:** biochar, modification, adsorption, phosphate, sodium alginate, recyclable

## Abstract

Although ball milling is effective for biochar modification with metal oxides for efficient phosphate removal, the recyclability of the adsorbent as well as the precursors for modification, still need to be optimized. Herein, a magnesium-modified biochar was first prepared with the precursor of MgCl_2_·6H_2_O through the solvent-free ball milling method. After that, recyclable biochar beads were fabricated with the introduction of sodium alginate and Fe_3_O_4._ The beads were proved to have excellent adsorption performance for phosphate with a saturated capacity of 53.2 mg g^−1^, which is over 12 times higher than that of pristine biochar beads. Although the particle size reduction, surface area, and O-containing group increments after milling are beneficial for adsorption, the remarkable promotion in performance should mainly result from the appropriate formation of magniferous crystals on biochar, which greatly accelerates the electrostatic interactions as well as precipitation for adsorption. The beads also exhibited excellent magnetism-driven recyclability, which greatly avoids secondary contamination and broadens the application field of the adsorbent.

## 1. Introduction

Water pollution, especially eutrophication caused by agricultural and industrial phosphate emissions, is drawing intensive attention all over the world. Excessive phosphate can stimulate the rapid growth of aquatic organisms, a series resulting in aquatic ecosystem problems [1,2]. Therefore, effective technologies for phosphate removal are urgently required. Adsorption is one of the most efficient ways of phosphate removal with the merits of easy operation and moderate cost; however, it is still highly demanding to develop an adsorbent with both adsorption and economic efficiency [3,4].

Recently, environmentally friendly biochar has been promoted as a cost-effective and efficient adsorbent, which is mainly produced by pyrolysis of prevalent waste biomass under oxygen-limited conditions [5]. These carbon-rich porous solids possess large surface areas and a significant density of O-containing functional groups, which are beneficial for pollutant adsorption. Until now, biochar has been widely used in the adsorption of various kinds of pollutants such as organics [6,7] and heavy metal ions [8]; however, its adsorption performance on phosphate is usually disillusionary due to electrostatic repulsion and limited precipitation with the adsorbate [9]. Therefore, surface modification is necessary for improving the phosphate adsorption performance of biochar, especially via Mg, Ca, and La impregnation [9]. The strong precipitation between phosphate and metal ions can increase the chemical adsorption of the adsorbate. However, traditional impregnation methods, such as dipping or filtration, are usually accomplished with the usage of metal salt solutions [10,11], which inevitably results in not only a waste of chemical solutions but also time and energy consumption [12].

Physical and mechanical treatment, especially ball milling, is also believed to be effective for biochar modification, which is far from the consumption of chemicals. The physical, mechanical process can not only introduce metal oxides on the surface of biochar but also reduce the particle size and increase the surface activities. Therefore, the capacity of the adsorbents can be increased in two ways for phosphate adsorption: (a) the introduced metal oxides are expected to increase the electrostatic attraction and precipitation with the adsorbates, and (b) the reduced particle size and increased surface activities are believed to enhance the active sites for adsorption. Till now, various metal oxides, such as CaO [13] and MgO [14], have been successfully introduced into biochar through ball milling. In particular, Xu et al. [14] recently prepared MgO-modified biochar by pyrolyzing the co-milled composites of biomass and Mg(CH_3_COO)_2_·4H_2_O, which greatly avoids extra energy loss as the biochar and the metal oxides can be formed at the same time. However, these milled composites usually exhibit good dispersion in water with their nano sizes, thus may result in secondary contamination without the property for easy separation and collection [15]. Meanwhile, it is preferable to utilize many common Mg precursors for replacing Mg(CH_3_COO)_2_·4H_2_O.

In this work, the quite common magnesium salt of MgCl_2_·6H_2_O was first utilized for biomass modification with a solvent-free ball milling process, and then the composites were pyrolyzed to prepare modified biochar. This solvent-free method is more eco-friendly and easier to operate than traditional impregnation ways. For better collecting the exhausted adsorbents from water, magnetic bead adsorbent was further prepared by compositing the modified biochar with sodium alginate and Fe_3_O_4_ through ionotropic gelation, which could avoid the secondary pollution caused by powder adsorbent. Sodium alginate will cross-link to form a network gel when it encounters Ca^2+^ (as can be seen in Figure S1 [16]); thus, MgBC and Fe_3_O_4_ will be encased in a network structure. The preparation conditions for the adsorbents were first pre-screened, and the physico-chemical properties of these adsorbents were examined carefully. The adsorption performance, as well as the mechanism of the resultant adsorbents, was also assessed and discussed.

## 2. Materials and Methods

### 2.1. Materials

The powder of maize straws was obtained from local suppliers. Magnesium chloride hexahydrate (MgCl_2_·6H_2_O, AR) and sodium hydroxide (NaOH, AR) were provided by Xilong Science Co., Ltd. (Guangdong, China). Ascorbic acid (AS, >99.0%) and iron (II, III) oxide (Fe_3_O_4_, 97%) were provided by Aladdin (Shanghai, China). Ammonium molybdate ((NH_4_)_2_MoO_4_, AR), L-antimony potassium tartrate (C_8_H_4_K_2_O_12_Sb_2_·xH_2_O, 99%), and potassium dihydrogen phosphate (KH_2_PO_4_, >99.5%) were provided by Macklin (Shanghai, China). Sulfuric acid (H_2_SO_4_, AR), sodium alginate (SA, CP), and anhydrous calcium chloride (CaCl_2_, AR) were supplied by Sinopharm Chemical Reagent Co., Ltd. (Shanghai, China). All chemicals were used without further purification. Deionized water was applied in all experiments.

### 2.2. Preparation of Magnesium-Modified Biochar

**Figure 1:**
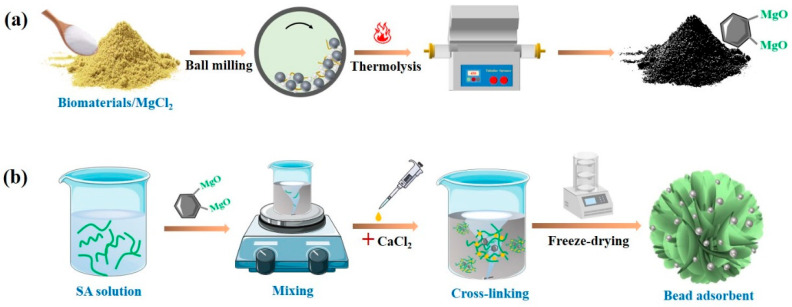
Schematic diagrams for the (**a**) MgBC and (**b**) bead preparation.

A solvent-free process was promoted here for magnesian biochar preparation, and the experimental procedure is shown in Figure 1a. To be specific, the straw powder (2 g) was first ball milled with a designated amount of MgCl_2_·6H_2_O at 300 rpm with a planetary ball mill (YXQM-0.4 L). The weight ratios between the precursors and the milling time were varied to seek the optimal preparation conditions. The magnesium-modified biomass was then pyrolyzed in an oxygen-limited environment with different temperatures for 5 h, and the modified biochar was named MgBC. The heating rate was set as 7.5 °C min^−1^.

### 2.3. Preparation of Bead Adsorbents

The as-obtained MgBC was incorporated into SA for bead preparation. The schematic diagram of the procedure is illustrated in Figure 1b. SA solution was first prepared by dissolving SA (1 g) in water (50 mL). After that, various amounts of MgBC were added and evenly dispersed in the SA solution with magnetic stirring. The suspension was then added dropwise to the magnetic-stirred CaCl_2_ solution (0.2 mol L^−1^, 200 mL) for preparing hydrogel beads by ionotropic gelation [16,17]. After stirring for 5 h, the as-obtained beads were washed three times with deionized water to remove excess Ca^2+^ ions. Afterwards, the samples were frozen and dried in a lyophilizer (Christ-Alpha 2-4 LSCplus) at −80 °C for 24 h. The obtained samples were named SA-MgBC-X, where X represents the mass (g) of the introduced MgBC. To further improve the recyclability of the beads, Fe_3_O_4_ (1 g) was also introduced with the addition of MgBC. The corresponding alginate beads were named FeSA-MgBC.

### 2.4. Characterizations

The Fourier transformation infrared spectra (FT-IR) of the samples were characterized by a Nicolet iS50 infrared spectrometer (Thermo Fisher Scientific, Waltham, MA, USA) with the attenuated total reflection mode (ATR). The X-ray diffraction (XRD) patterns were also characterized with a wide-angle X-ray diffractometer (D2 Phaser, Bruker, Germany) under Cu Kα radiation (λ = 1.5418 Å). The voltage and current of the generator were set at 30 kV and 10 mA, respectively. An EVO LS 10 scanning electron microscope (SEM, Zeiss, Baden-Wertenburg, Germany) was used to examine the morphologies of the samples under the accelerated voltage of 10 kV. A magnetometer (MPMS XL-7, Quantum Design, Caledonia, IL, USA) with a versalab system was used to characterize the magnetism of the adsorbents at room temperature with the magnetic field ranging from −20 to 20 kOe. The specific surface area was obtained using the Brunauer–Emmett–Teller method (BET, Autosor-IQ, Quantachrome, Boynton Beach, FL, USA). Chemical compositions of the adsorbents were analyzed with energy-dispersive X-ray spectroscopy (EDS, SUPRA55, Zeiss, Jena, Germany).

### 2.5. PO_4_-P Adsorption

Different adsorbents (10 mg) were added to the KH_2_PO_4_ solution (10 mg L^−1^, 50 mL) to explore their phosphate (PO_4_-P) adsorption performance. The solution was then placed in a shaker (ISRDV1) at 25 °C for 24 h to achieve adsorption equilibrium. Concentrations of phosphate before and after adsorption were determined by the ammonium molybdate spectrophotometry method. Specifically, orthophosphate in the solution can be reduced to phosphomolybdic acid with the reducing agent of ascorbic acid and then form a blue complex with the addition of ammonium molybdate and potassium antimony oxytartrate [18]. Color comparison of phosphate solution before and after adsorption after chromogenic reaction can be seen in Figure S2. UV-vis absorption spectrophotometer (NanoDrop ONE^C^, Thermo Fisher Scientific, Waltham, MA, USA) was used to characterize the concentrations of phosphate by comparing the UV absorbance at the wavelength of 700 nm. Notably, the adsorbent was separated from the solution by filtering through a nylon filter with a pore size of 0.22 µm after adsorption. The removal capacity (*q_e_*, mg g^−1^) was calculated with Equation (1):

(1) $$q_{e}=\frac{(C_{0}-C_{e})V}{m},$$

where *C*_0_ and *C_e_* are the PO_4_-P concentrations before and after adsorption (mg L^−1^). *V* is the volume of the solution (L), and *m* is the mass of the adsorbent (g). All of the adsorption experiments were performed three times without pH adjustment except for studying the effect of pH on adsorption.

In order to further explore the adsorption process of phosphate by the adsorbent, adsorption kinetics, isotherms, and thermodynamics were carried out. The pseudo-first-order (Equation (2)) and pseudo-second-order (Equation (3)) models were applied to analyze the adsorption kinetics of FeSA-MgBC-3:

(2) $$q_{t}=q_{e}\left(1-e^{-k_{1}t}\right),$$

(3) $$\frac{t}{q_{t}}=\frac{1}{k_{2}q_{e}{}^{2}}+\frac{t}{q_{e}},$$

where *k*_1_ (min^−1^) and *k*_2_ (g mg^−1^ min^−1^) correspond to the rate constants of the corresponding models, *q_t_* (mg g^−1^) and *q_e_* (mg g^−1^) represents the amount of PO_4_-P adsorbed at time *t* (min) and equilibrium.

The Langmuir (Equation (4)) and Freundlich (Equation (5)) models were applied to describe the isothermal adsorption process:

(4) $$q_{e}=\frac{K_{L}q_{L}C_{e}}{1+K_{L}C_{e}},$$

(5) $$q_{e}=K_{F}C_{e}{}^{1/n},$$

where *q_e_* (mg g^−1^) is the amount of PO_4_-P adsorbed at equilibrium and *q_L_* represents the adsorption capacity of PO_4_-P at saturation; *n*, *K_L_* (L mg^−1^), and *K_F_* ((mg g^−1^) (L mg^−1^)^1/n^) are constants for the corresponding models.

The relevant parameters of thermodynamic adsorption can be calculated with Equations (6) and (7):

(6) $$\ln\left(K_{d}\right)=\frac{T\Delta S{}^{\circ}-\Delta H{}^{\circ}}{RT},$$

(7) $$\Delta G{}^{\circ}=-RTln\left(K_{d}\right),$$

where *K_d_* is the equilibrium constant (L g^−1^), which can be obtained by plotting ln (*q_e_/C_e_*) as a function of *q_e_*, *R* is the universal gas constant (8.314 J mol^−1^ K^−1^), and *T* is the system temperature (K).

### 2.6. Cyclic Performance Test

In order to determine the reusability of FeSA-MgBC, 5 cycles of adsorption–desorption experiments were carried out. The adsorption process was consistent with the previous experimental conditions. For desorption, beads soaked in 1 mol L^−1^ NaOH solution were used and then washed to neutral after desorption. Then, the regenerated adsorbent was used for adsorption under the same experimental conditions.

## 3. Results and Discussion

### 3.1. Pre-Screening the Conditions for Adsorbent Preparation

**Figure 2:**
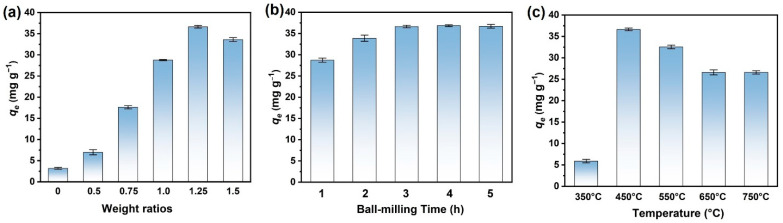
The adsorption capacities of MgBCs with different (**a**) mass ratios of MgCl_2_·6H_2_O and biochar, (**b**) ball-milling time, and (**c**) pyrolysis temperatures.

The optimal conditions for MgBC preparation were explored by comparing the adsorption capacities of the sorbents with various preparation conditions (different mass ratios of precursors, durations for ball milling, and temperatures for pyrolysis). The mass ratio of the precursors was first changed from 0 to 1.5 with fixed milling time (3 h) and pyrolysis temperature (450 °C). As shown in Figure 2a, pristine biochar exhibited quite limited adsorption capacity (~3.2 mg g^−1^) for PO_4_-P. Therefore, it was necessary to modify the biochar for adsorption improvements. Here, biomass was pyrolyzed after ball milling with magnesium salt to improve the metal discharge and anionic pollutant adsorption capacities of the biochar [9]. After introducing the Mg precursor, the capacities of the adsorbents for phosphate adsorption were dramatically increased. In addition, the adsorption performance was greatly correlated with the mass ratios between the precursors. As shown in Figure 2a, the adsorption capacity was increased with the increment of the mass ratios and finally reached ~36.6 mg g^−1^ as the ratio came to 1.25. The capacity was, however, further decreased after the ratio increased over 1.25. As revealed by FT-IR and XRD patterns (Figure S3a,b), the introduced Mg^2+^ finally converted to MgO as the characteristic peaks centering at ~500 cm^−1^ and 42.9° matched well with the standard data (PDF number 45-0946) [19]. Meanwhile, higher ratios led to enhanced characteristic peaks, representing that more MgO was formed. As observed from the SEM images of pristine biochar and MgBC (Figure S4), the surfaces of modified biochar were coated with some lamellar crystals, which should be ascribed to the introduced MgO. Meanwhile, the crystal increased and aggregated with the ratio. Zeta potentials of the MgBC were also measured with the results in Table 1. It can be seen in Table 1 that the surface potential of the magnesium-modified biochar was positive, which is conducive to promoting the electrostatic attraction between the biochar and the phosphate anion so as to improve the adsorption performance of phosphate, while the negative surface potential of the original biochar will generate electrostatic repulsion with the phosphate anion, thus hindering adsorption. The existence of MgO greatly promotes the potential; however, no obvious difference was observed with an increased amount of MgO, which indicates that the electrostatic interactions may not change with increased loading of MgO. Therefore, adsorption promotion should originate from the surface precipitation of phosphate with an increased amount of Mg^2+^ released from the adsorbents [20]. The BET results (Table 1) showed that the specific surface area increases with the ratio at first. However, a slight decrease was observed as the ratio changed from 1.25 to 1.5. That means aggregation and pore-clogging strengthened with an excess amount of MgO on biochar, which led to a recession of adsorption sites for complexation. Therefore, the mass ratio was further settled at 1.25.

The milling durations were then regulated. As shown in Figure 2b, adsorption was promoted with prolonged ball milling durations, and it reached the saturated value of 36.6 mg g^−1^ with a duration of 3 h. Further increment of the milling time had nearly no effect on adsorption. The XRD spectra confirmed the saturation of the deposited MgO after milling for 3 h as the intensity of the characteristic peak of MgO nearly had no change with further prolonged duration (Figure S5). Therefore, it was believed that the biochar was saturated and deposited with Mg^2+^ after 3 h milling. The BET results (Table 1) showed that the specific surface area increased as the grinding time changed from 1 to 3 h, which resulted from the generated high energy shear forces. It had a slight decrease with a 5 h process, which may have resulted from the partial collapse of the matrix. Therefore, the adsorption capacity reached the maximum value at 3 h.

Pyrolysis temperature for adsorbent preparation was also found to have a vital effect on adsorption. As shown in Figure 2c, MgBC pyrolyzed at 350 °C has quite a low adsorption capacity of only ~5.9 mg g^−1^, which is close to the capacity of the biochar pyrolyzed at 450 °C without magnesian modification. That means the temperature of 350 °C is not high enough to transform Mg^2+^ to MgO, which is confirmed by the XRD results (Figure S6a). As the temperature reached 450 °C, the capacity increased dramatically. However, further increment of pyrolysis temperature had a negative effect on PO_4_-P adsorption. FT-IR spectra (Figure S6b) showed that the oxygen-containing functional groups in the adsorbents gradually disappeared with the increase in temperature (as evidenced by the C=O, C-O stretching vibration peaks). However, MgO was maintained with temperature as high as 750 °C. As shown in Figure S7, the as-formed MgO was aggregated into clusters after pyrolyzing at 550 and 650 °C, which is no longer lamellar. The BET results showed that the surface area of the composites increases with the temperature below 550 °C, which is due to the fact that more substances can be volatilized to create more pore structure. However, a much higher temperature of >550 °C may result in the collapse of the pore, thus greatly reducing the surface areas. Therefore, we speculated that although the sample pyrolyzed at 550 °C has the highest specific surface area, the aggregated MgO, as displayed in microscopic images, greatly retarded the release of Mg^2+^ for precipitation with phosphate, thus leading to decreased adsorption capacities. This recessive performance was also observed in the literature [21]. After pre-screening the conditions, the optimal MgBC was finally prepared with a mass ratio of 1.25, a milling time of 3 h, and a pyrolysis temperature of 450 °C. The N_2_ adsorption–desorption isotherms and pore size distribution of MgBC under optimal preparation conditions are shown in Figure S8. It can be seen from the figure that the isotherm belongs to type II and exhibits the H3 hysteresis loop. In addition, the pore size distribution is mainly in the range of 3.3–5.4 nm, indicating that MgBC is mesoporous with an average pore size of 4.5 nm. Powder adsorbent MgBC under this preparation conditions was further utilized in subsequent experiments.

**Figure 3:**
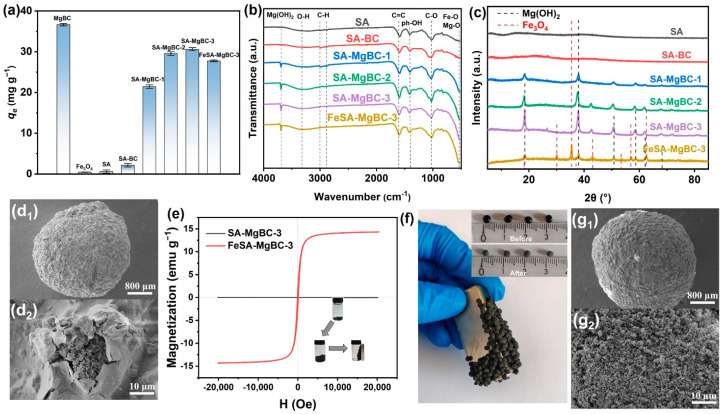
Characterizations of bead adsorbents. (**a**) *q_e_* of bead adsorbents for PO_4_-P, (**b**) FT-IR and (**c**) XRD spectra of bead adsorbents, (**d**) SEM images of SA-MgBC-3, (**e**) magnetic hysteresis loops of SA-MgBC-3 and FeSA-MgBC-3 at room temperature, the inset is the photograph showing its magnetic separability, (**f**) macroscopic photograph of the magnetic bead adsorbent attracted to the magnet, the inset is the photograph showing changes of gel microspheres before and after lyophilization, and (**g**) SEM images of FeSA-MgBC-3.

However, it is difficult to collect the powder adsorbent from the water after use, which will cause secondary contamination. Therefore, the powder of MgBC was then composited with SA for gel beads preparation. The beads can be separated from water more easily, thus facilitating their applications in various fields, such as column-type filtration. To further improve the recyclability, the beads were also modified by introducing magnetic Fe_3_O_4_. Various weights of MgBC with a fixed amount of SA (1 g) were first applied. SA beads without MgBC or with unmodified biochar (1 g) were also prepared for comparison. It was found that SA beads without the addition of biochar had almost no adsorption on PO_4_-P (Figure 3a). Therefore, the addition of SA was expected to reduce the adsorption capacity of PO_4_-P when an equal amount of powder and bead adsorbents are used. As shown in Figure 3a, the adsorption was only slightly increased with the introduction of unmodified biochar, which probably originated from the quite limited adsorption capacity of biochar (Figure 2a). The introduction of MgBC otherwise dramatically increased the capacity even though the capacity was still slightly lower than that of pristine MgBC. More MgBC leads to higher capacity. However, the adsorption performance of the adsorbents with a ratio of 3 was only slightly higher than that with a ratio of 2, representing that adsorption may not be further promoted with an excess amount of MgBC. Meanwhile, it was found that excess amounts of magnesium-modified biochar cannot be dispersed in SA solution any more. Therefore, SA-MgBC-3 was used for subsequent experiments. The FT-IR spectra showed the appearance of the peak of Mg(OH)_2_ at ~3700 cm^−1^ after gelation, which revealed the transformation of loaded MgO to Mg(OH)_2_ after reacting with SA solution (Figure 3b) [22]. XRD results in Figure 3c also confirmed the existence of Mg(OH)_2_, which coincides with standard peaks (PDF number 44-1482). The SA-MgBC-3 (Figure 3d) has a quite rugged surface with abundant macropores, thus the transfer of adsorbate into sorbents may not be hindered. In consideration of the limited PO_4_-P adsorption capacity of pristine SA, adsorption was mainly carried out with SA-MgBC-3.

Based on the preparation of hybrid beads, magnetism was further introduced. FT-IR (Figure 3b, the newly formed vibration peak of Fe-O at ~543 cm^−1^) and XRD (Figure 3c, matched with PDF number 01-019-0629) patterns both confirmed the successful introduction of Fe_3_O_4_. Meanwhile, it was found that the adsorption capacity was slightly decreased (~9.3%) as Fe_3_O_4_ nearly had no adsorption on PO_4_-P (Figure 3a). Considering that the addition of 1 g Fe_3_O_4_ did not significantly reduce the adsorption performance, this may have resulted from the positive potential on the surface of Fe_3_O_4_ promoting electrostatic attraction between phosphate anions, thus contributing to the adsorption capacity to a certain extent. Moreover, the magnetism was obtained as revealed by the magnetic hysteresis loop (Figure 3e). Magnetic saturation achieved 14.3 emu g^−1^, which is quite useful for adsorbent collection. As shown in the inset in Figure 3e, the lyophilized particles swelled in the solution and can be further magnetically attracted by a magnet. Thus, the beads attached to the precipitate after precipitation can be easily separated from the water and avoid the possibility of secondary pollution, and the beads, after adsorption, can be used as fertilizer. Figure 3f also confirms the strong attraction between magnetic bead adsorbent and the magnet, implying easier collection of the adsorbents. The SEM images of FeSA-MgBC-3 are depicted in Figure 3g. Compared with that of SA-MgBC-3, no obvious change was observed, implying the adsorption of the hybrids was also mainly guided by the interactions between phosphate ions and MgBC. The EDS results (Figure S9 and Table S1) confirmed the existence of the Fe element in FeSA-MgBC-3. In addition, considerable amounts of Ca and Mg elements were both observed in the two samples, implying the successful preparation of the bead hybrids.

### 3.2. Adsorption Kinetics, Isotherms and Thermodynamics of the Bead Adsorbents

#### 3.2.1. Adsorption Kinetics

**Figure 4:**
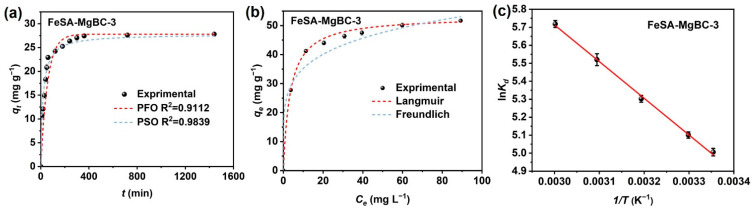
Adsorption (**a**) kinetics, (**b**) isotherms, and (**c**) thermodynamics of PO_4_-P on FeSA-MgBC-3.

The adsorption process of PO_4_-P on FeSA-MgBC-3 is shown in Figure 4a. A rapid adsorption stage was observed in the initial 2 h, in which 55.6% adsorbate was adsorbed. The fast mass transfer process mainly originated from the electrostatic attraction between the positively charged adsorbent surface and the negatively charged PO_4_^3−^, as well as the complexation between the rapidly released Mg^2+^ from the surface of the adsorbents and the adsorbates [23]. After that, adsorption became much slower, and equilibrium was finally achieved within 400 min, which may correspond to the subsequent slowly performed complexation process with sub-layer MgO. The pseudo-first-order and pseudo-second-order models were applied to analyze the adsorption kinetics of FeSA-MgBC-3. The corresponding fitted curves and parameters are depicted and listed in Figure 4a and Table 2, respectively. Adsorption was better described with the pseudo-second-order kinetic model (*R*^2^ > 0.98), implying that adsorption is mainly controlled by chemisorption [24,25].

#### 3.2.2. Adsorption Isotherms

In addition, isothermal adsorption experiments were also performed. The Langmuir and Freundlich models were applied for fitting. As shown in Figure 4b and Table 3, adsorption was better fitted with the Langmuir (*R*^2^ = 0.999) than Freundlich isotherm models (*R*^2^ = 0.982), which is consistent with reported MgO-impregnated magnetic biochar [26]. This indicated that the maximum phosphate adsorption capacity was 53.2 mg g^−1^.

In addition, in order to compare the adsorption properties of adsorbents prepared in this work with those reported in related studies, the saturated phosphate adsorption capacity (*q_m_*) calculated by the Langmuir model in some related studies is listed in Table 4. Although a few related works have comparable or more remarkable capacities for phosphate, these adsorbents are usually prepared with the usage of solvent and are also hard to be separated. Therefore, the adsorbent prepared in this work was prominent in phosphate adsorption.

#### 3.2.3. Adsorption Thermodynamics

To further determine the thermodynamic parameters of PO_4_-P adsorption onto FeSA-MgBC-3, experiments were carried out at five different temperatures from 25 to 60 °C. The parameters, including entropy change (Δ*S*°, J mol^−1^ K^−1^), enthalpy change (Δ*H*°, kJ mol^−1^), and standard Gibbs free energy change (Δ*G*°, kJ mol^−1^) can be calculated with Equations (6) and (7). The plot of ln*K_d_* versus 1/*T* for PO_4_-P adsorption is provided in Figure 4c with the corresponding parameters listed in Table 5. Negative Δ*G*° decreases with the increase in temperature, which indicates adsorption is spontaneous [27]. Positive Δ*H*° implies the endothermic nature of adsorption, and the adsorption capacity can be promoted with the increase in temperature [28]. Positive Δ*S*° represents the adsorbate-adsorbent interface that has increased randomness during adsorption [29].

### 3.3. Effect of pH Values on Adsorption Performance

**Figure 5:**
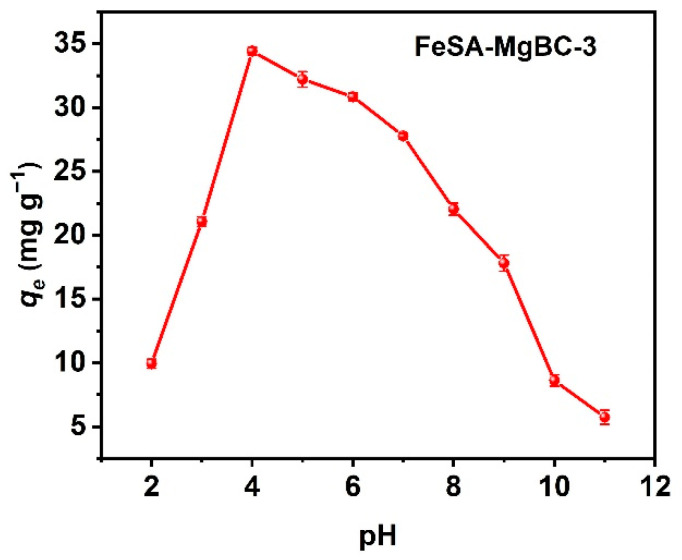
*q_e_* for PO_4_-P at different pH values of FeSA-MgBC-3.

The effect of pH on the adsorption of FeSA-MgBC-3 was evaluated by changing the value from 2 to 11. As shown in Figure 5, the adsorption capacity of PO_4_-P increased with the pH, ranging from 2 to 4, and reached the optimal value of 34.4 mg g^−1^ at the pH of 4. Further increment of pH leads to a decreased adsorption capacity. Varied adsorption capacity should result from the changed forms of phosphate at different pH values [30,31]. As pH < 2, the predominant form of phosphorus is H_3_PO_4_, which is hard to generate electrostatic attraction with the adsorbent. As the pH value increases to 4, H_2_PO_4_^−^ is gradually generated. The loaded MgO and Mg(OH)_2_ on the biochar will be protonated to MgOH^+^ under acidic conditions, thus resulting in the positively charged adsorbents. Strong electrostatic attraction between the adsorbent and H_2_PO_4_^−^ took place, thus greatly promoting adsorption [32]. With the further increment of pH value, H_2_PO_4_^−^ gradually transformed into HPO_4_^2−^ and PO_4_^3−^, while MgO and Mg (OH)_2_ were less protonated or even negatively charged, thus resulting in reduced adsorption. Therefore, the electrostatic interactions were believed to have a vital effect on adsorption.

### 3.4. Adsorption Mechanisms

**Figure 6:**
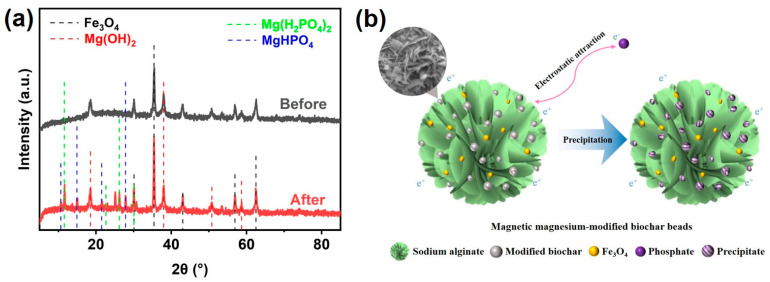
(**a**) XRD spectra of FeSA-MgBC-3 before and after adsorption. (**b**) Schematic representation of the mechanism of interaction with phosphate removal.

In order to further clarify the mechanism of phosphorus adsorption on FeSA-MgBC-3, the XRD characteristic of the adsorbent before and after phosphorus adsorption was presented (Figure 6a). Additional characteristic peaks, which belong to Mg(H_2_PO_4_)_2_ (PDF number 40-0066) and MgHPO_4_ (PDF number 46-0375), were observed after adsorption. This indicated that the precipitation between Mg ions and phosphate ions was taking place, thus greatly facilitating the adsorption performance of the adsorbent.

The formation of MgO on MgBC was expected to follow the reaction sequence of MgCl_2_·6H_2_O → MgCl_2_ → Mg(OH)_2_ → MgO with gas forms of HCl and H_2_O produced and swept out by the N_2_ airflow [33]. Then, MgO reacted with H_2_O and converted to Mg(OH)_2_ during the production of the beads. Subsequently, the beads were applied for phosphate adsorption. At the beginning of adsorption, phosphate was electrostatically attracted with surface-anchored MgO and Mg(OH)_2_ as Mg(OH)_2_ can be protonated into MgOH^+^. Upon contact with the magniferous crystals, the phosphate begins to complex and precipitate with the following process [26]:

$${\mathrm{MgOH}}^{+}+2H_{2}{\mathrm{PO}}_{4}^{-}\;\overset{\;}{\to }\;\mathrm{Mg}{\left(H_{2}{\mathrm{PO}}_{4}\right)}_{2}+{\mathrm{OH}}^{-},\;{\mathrm{MgOH}}^{+}+{\mathrm{HPO}}_{4}^{2-}\;\overset{\;}{\to }{\;\mathrm{MgHPO}}_{4}+{\mathrm{OH}}^{-}.$$

Precipitation was carried out continuously, resulting in the formation of Mg(H_2_PO_4_)_2_ and MgHPO_4_. Electrostatic attraction is quite important as it can drive and accelerate precipitation. However, even though pristine biochar was negatively charged, no complexation and precipitation occurred during the adsorption, thus resulting in quite limited phosphorus adsorption. Thus, the precipitation of PO_4_-P by metal oxides in biochar was the main mechanism for phosphate removal, which was consistent with the reported literature [34]. At the same time, considering that the adsorption of phosphate is greatly related to the active sites for releasing Mg^2+^, the agglomeration of MgO and Mg(OH)_2_ on MgBC with higher conversion temperatures will inevitably lead to the recession of adsorption. Therefore, the appropriate formation of magniferous crystals was the key to the adsorption improvement. The schematic representation of the mechanism of interaction with phosphate removal is shown in Figure 6b.

### 3.5. Cyclic Adsorption Experiments

**Figure 7:**
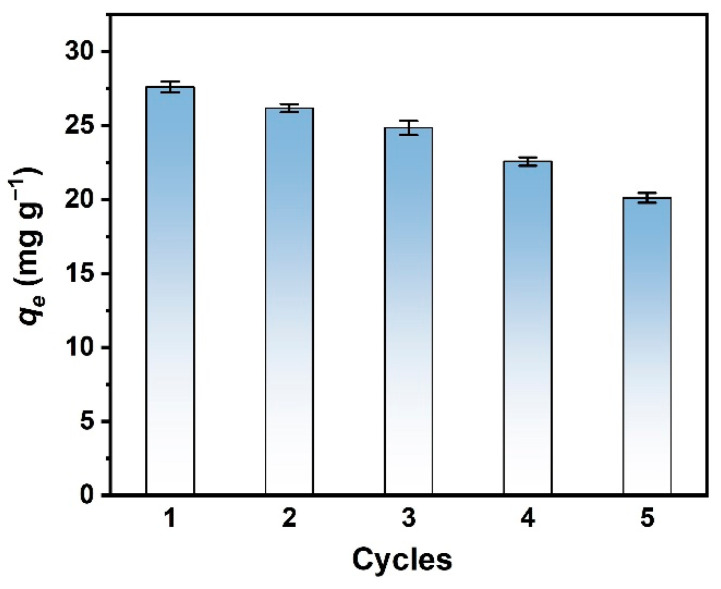
*q_e_* of FeSA-MgBC-3 at different cycles.

The exhausted beads were regenerated and recycled with NaOH solution. As shown in Figure 7, the equilibrium adsorption capacity of the adsorbent on PO_4_-P was 72.9% of the original capacity after five cycles. The declined performance should be ascribed to the loss of adsorption sites with strongly trapped adsorbates [14]. However, the decline rate of the capacity of the adsorbent was much lower than that of biochar adsorbents reported in the literature [35], indicating the adsorbent promoted in this work is favorable for recycling.

## 4. Conclusions

In this work, recyclable magnesium-modified biochar (MgBC) beads were successfully fabricated with MgCl_2_·6H_2_O, sodium alginate, and Fe_3_O_4_. MgBC was produced with a solvent-free ball milling method, thus greatly avoiding chemical contamination. The beads had excellent adsorption performance for PO_4_-P with a saturated adsorption capacity of 53.2 mg g^−1^, which is >12 times higher than that of pristine biochar beads. Adsorption was mainly driven by electrostatic interactions as well as precipitation. Although the reduction of particle size and the increment of surface areas and O-containing groups after grinding were beneficial for adsorption, proper formation of magnetic crystals on biochar was proved as the key to adsorption improvement. The beads also exhibited excellent magnetism-driven recyclability, thus greatly avoiding secondary contamination.

## Figures and Tables

**Table 1:** BET and Zeta potentials of MgBCs with different preparation conditions.

Sample	BET Surface Area (m^2^ g^−1^)	Zeta Potential (mV)
BC	36.99	−15.62 ± 0.37
MgBC-450 °C-3 h-0.75	35.22	5.73 ± 0.47
MgBC-450 °C-3 h-1.25	134.95	8.45 ± 0.43
MgBC-450 °C-3 h-1.5	110.02	7.79 ± 0.96
MgBC-450 °C-1 h-1.25	67.74	7.04 ± 0.14
MgBC-450 °C -5 h-1.25	126.33	7.81 ± 0.54
MgBC-550 °C-3 h-1.25	203.67	4.93 ± 0.30
MgBC-650 °C-3 h-1.25	153.91	4.73 ± 0.78

**Table 2:** Kinetic adsorption parameters in this study.

Kinetic Models	Parameters	FeSA-MgBC-3
Experimental	*q_e_* (mg g^−1^)	27.8
Pseudo-first-order	*q_e_* (mg g^−1^)	27.8
	*k_1_* (min^−1^)	0.018
	*R* ^2^	0.911
Pseudo-second-order	*q_e_* (mg g^−1^)	27.8
	*k_2_* (g mg^−1^ min^−1^) ×10^−3^	7.85
	*R* ^2^	0.984

**Table 3:** Parameters of adsorption isotherm models in this study.

Isotherm Models	Parameters	FeSA-MgBC-3
Langmuir	*q_L_* (mg g^−1^)	53.2
	*k_L_* (L mg^−1^)	0.292
	*R* ^2^	0.999
Freundlich	*n*	5.329
	*k_F_* ((mg g^−1^) (L mg^−1^)^1/n^)	22.86
	*R* ^2^	0.982

**Table 4:** *q_m_* of relevant adsorbents.

Materials	*q_m_* for P (mg g^−1^)	References
Magnetic magnesium-modified biochar beads	53.2	This work
Lanthanum loaded biochar	46.4	[3]
FeCl_3_-impregnated biochar	90.0	[10]
MgFe_2_O_4_-biochar-based lanthanum alginate bead	26.8	[17]
MgO-modified biochar from waste woody	29.2	[20]
Functionalizing biochar with Mg-Al and Mg-Fe layered double hydroxides	15.2	[25]
Slow pyrolyzed biochar from corncobs	10.9	[27]

**Table 5:** Thermodynamic parameters of adsorption in this study.

	***T* (K)**	**Δ*G*° (kJ mol** **^−1^)**	**Δ*H*° (kJ mol** **^−1^)**	**Δ*S*° (J mol^−1^ K^−1^)**
FeSA-MgBC-3	298.15	−12.37	16.96	98.37
	303.15	−12.86		
	313.15	−13.84		
	323.15	−14.83		
	333.15	−15.81		

## Data Availability

The data presented in this study are available upon request from the corresponding author.

## Supplementary Materials

The following supporting information can be downloaded at: https://www.mdpi.com/article/10.3390/nano13060966/s1. Figure S1: Schematic diagram of sodium alginate gel reaction. Figure S2: Color comparison of phosphate solution before and after adsorption after the chromogenic reaction. Figure S3: (a) FT-IR and (b) XRD spectra of MgBC with different mass ratios of MgCl_2_·6H_2_O and biochar. Figure S4: SEM images of different mass ratios of MgCl_2_·6H_2_O and biochar (a) 0, (b) 0.75, (c) 1.25, and (d) 1.5. Figure S5: XRD spectra of MgBC at different ball-milling times. Figure S6: (a) XRD and (b) FT-IR spectra of MgBC at different pyrolysis temperatures. Figure S7: SEM images of adding 2.5 g MgCl_2_·6H_2_O MgBC pyrolyzed at (a) 550 °C and (b) 650 °C. Figure S8. N_2_ adsorption–desorption isotherms and pore size distribution of MgBC under optimal preparation conditions. Figure S9: EDS results of SA-MgBC-3 and FeSA-MgBC-3. Table S1: EDS results of SA-MgBC-3 and FeSA-MgBC-3.
