# Supplementary material for: Recyclable Magnesium-Modified Biochar Beads for Efficient Removal of Phosphate from Wastewater
Source: Nanomaterials (Basel). 2023 Mar 7;13(6):966. doi: 10.3390/nano13060966 (PMC10051089; doi:10.3390/nano13060966)
Supplement: Supplementary file 1 [file nanomaterials-13-00966-s001.zip › nanomaterials-2238581-supplementary.pdf]

nanomaterials

Supplementary Materials

Recyclable Magnesium-Modified Biochar Beads for Efficient Removal of Phosphate from Wastewater

Biao Hu [1], Nina Yan [2], Zhiyu Zheng [2], Lei Xu [2], Hongde Xie [1,*] and Jingwen Chen [2,*]

[1] College of Chemistry, Chemical Engineering and Materials Science, Soochow University, Suzhou 215123, China
[2] Key Laboratory for Protected Agricultural Engineering in the Middle and Lower Reaches of Yangtze River, Institute of Agricultural Facilities and Equipment, Jiangsu Academy of Agricultural Sciences, Ministry of Agriculture and Rural Affairs, Nanjing 210014, China
* Correspondence: xiehongde@suda.edu.cn (H.X.); chenjingwen@jaas.ac.cn (J.C.)

1. Supplementary Figures and Tables

1.1. Supplementary Figures

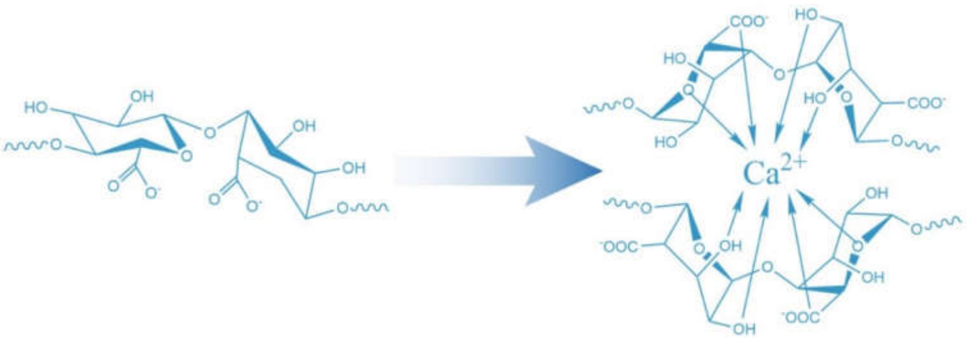

Figure S1. Schematic diagram of sodium alginate gel reaction.

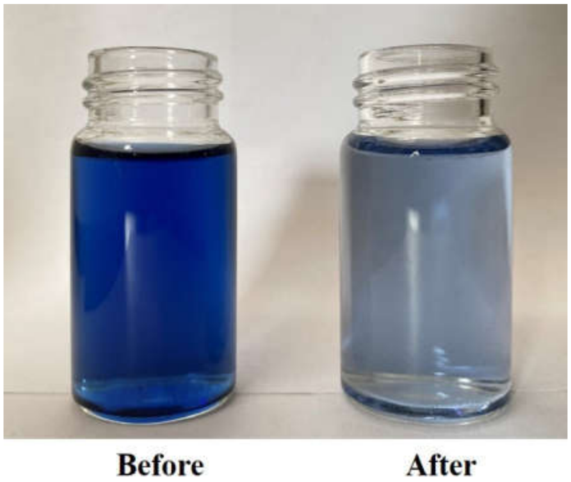

Figure S2. Color comparison of phosphate solution before and after adsorption after chromogenic reaction.

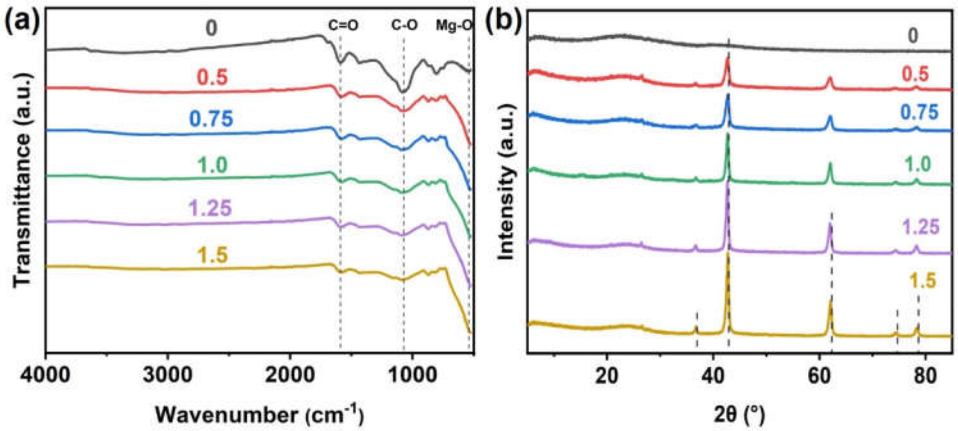

Figure S3. (a) FT-IR and (b) XRD spectra of MgBC with different mass ratios of $MgCl_2·6H_2O$ and biochar.

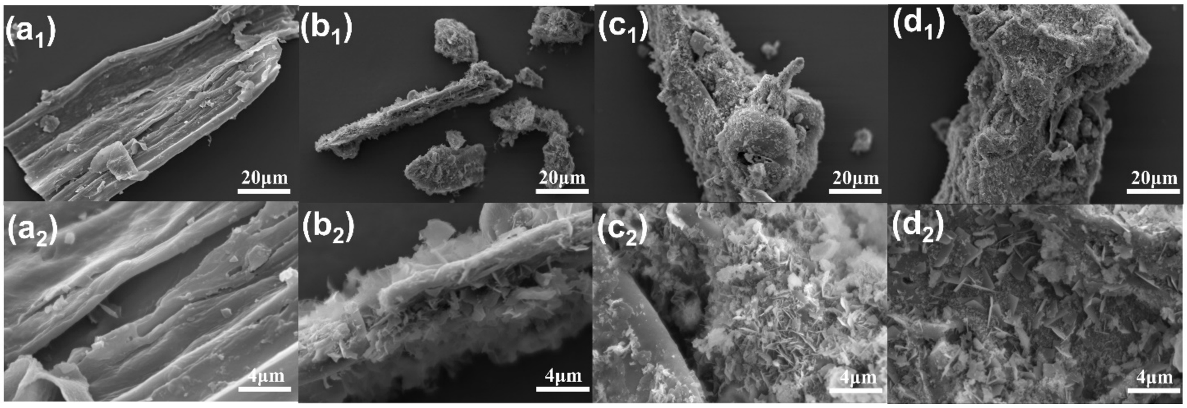

Figure S4. SEM images of different mass ratios of $MgCl_2·6H_2O$ and biochar (a) 0, (b) 0.75, (c) 1.25 and (d) 1.5.

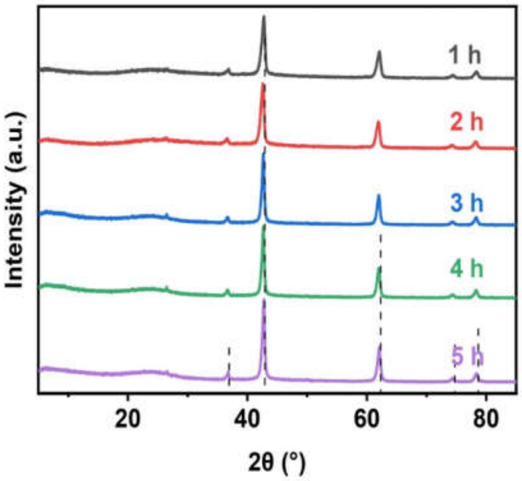

Figure S5. XRD spectra of MgBC at different ball-milling time.

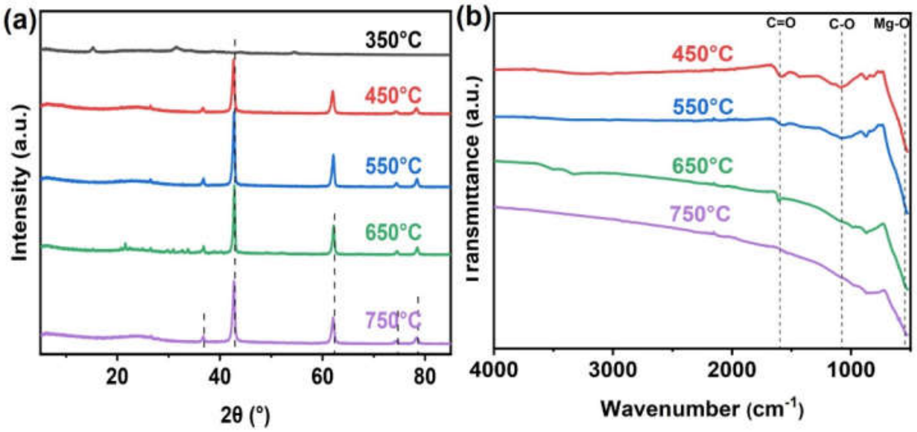

Figure S6. (a) XRD and (b) FT-IR spectra of MgBC at different pyrolysis temperature.

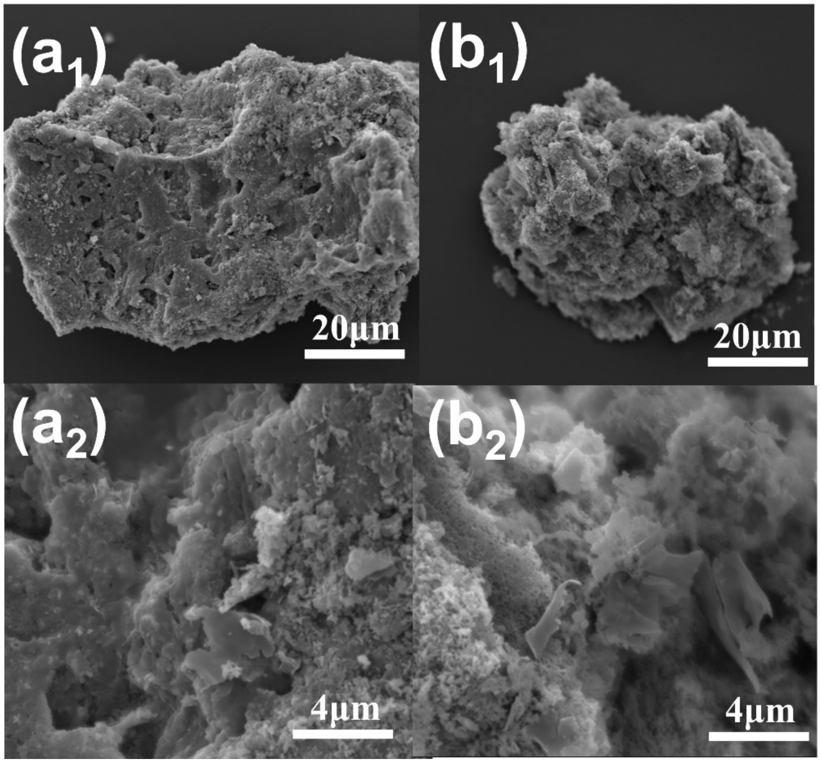

Figure S7. SEM images of MgBC pyrolyzed at (a) 550 °C and (b) 650 °C.

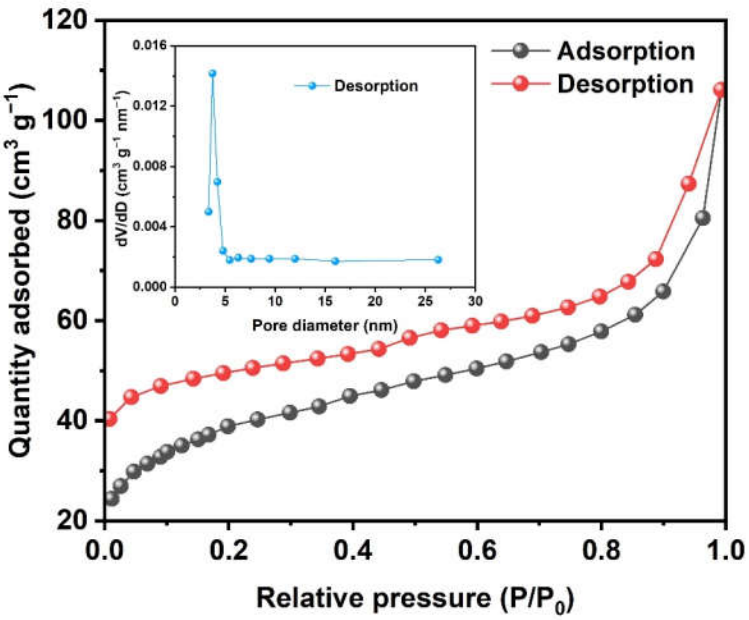

Figure S8. N_2 adsorption-desorption isotherms and pore size distribution of MgBC under optimal preparation conditions.

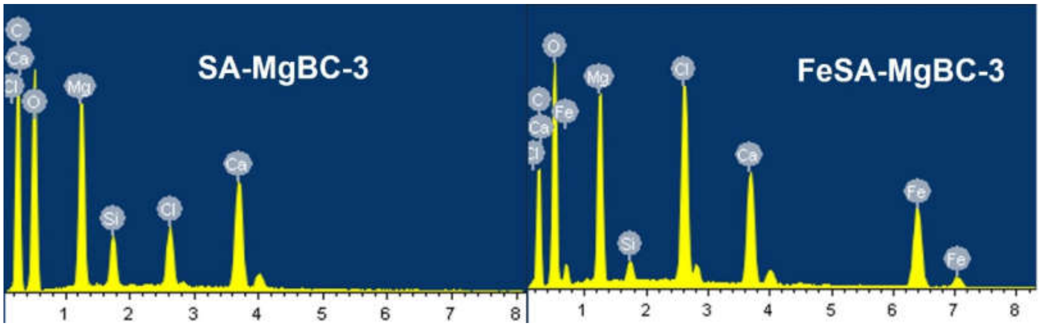

Figure S9. EDS results of SA-MgBC-3 and FeSA-MgBC-3.

1.2. Supplementary Tables

Table S1. EDS results of SA-MgBC-3 and FeSA-MgBC-3.

	Weight ratio (wt%)						
	C	*O*	*Mg*	*Ca*	*Cl*	*Si*	*Fe*
SA-MgBC-3	*40.42*	*42.72*	*6.66*	*6.04*	*2.53*	*1.63*	*0*
FeSA-MgBC-3	*35.30*	*38.70*	*6.83*	*5.73*	*1.64*	*0.63*	*11.17*
